# Implementation of type 1 diabetes genetic risk screening in children in diverse communities: the Virginia PrIMeD project

**DOI:** 10.1186/s13073-024-01305-8

**Published:** 2024-02-14

**Authors:** Kristin A. Guertin, David R. Repaske, Julia F. Taylor, Eli S. Williams, Suna Onengut-Gumuscu, Wei-Min Chen, Sarah R. Boggs, Liping Yu, Luke Allen, Lacey Botteon, Louis Daniel, Katherine G. Keating, Mika K. Labergerie, Tyler S. Lienhart, Jorge A. Gonzalez-Mejia, Matt J. Starnowski, Stephen S. Rich

**Affiliations:** 1 Department of Public Health Sciences, University of Virginia, 1300 Jefferson Park Avenue, 3182 West Complex, Charlottesville, VA 22903 USA; 2 Department of Public Health Sciences, UConn School of Medicine, UConn Health, 263 Farmington Avenue, MC 6325, Farmington, CT 06030 USA; 3 Department of Pediatrics, Division of Pediatric Diabetes & Endocrinology, University of Virginia, UVAHealth, 1204 W Main Street, 6th Floor, Charlottesville, VA 22903 USA; 4 Department of Pathology, Division of Medical Genetics, UVAHealth, University of Virginia, 21 Hospital Drive, Charlottesville, VA 22903 USA; 5 Center for Public Health Genomics, University of Virginia, 1335 Lee Street, 3235 West Complex, Charlottesville, VA 22903 USA; 6 Barbara Davis Center for Diabetes, University of Colorado Anschutz Medical Campus, 1774 Aurora Court, Suite A140, Aurora, CO 80045 USA

**Keywords:** Community genetic screening, Type 1 diabetes, Diverse populations

## Abstract

**Background:**

Population screening for risk of type 1 diabetes (T1D) has been proposed to identify those with islet autoimmunity (presence of islet autoantibodies). As islet autoantibodies can be transient, screening with a genetic risk score has been proposed as an entry into autoantibody testing.

**Methods:**

Children were recruited from eight general pediatric and specialty clinics across Virginia with diverse community settings. Recruiters in each clinic obtained informed consent/assent, a medical history, and a saliva sample for DNA extraction in children with and without a history of T1D. A custom genotyping panel was used to define T1D genetic risk based upon associated SNPs in European- and African-genetic ancestry. Subjects at “high genetic risk” were offered a separate blood collection for screening four islet autoantibodies. A follow-up contact (email, mail, and telephone) in one half of the participants determined interest and occurrence of subsequent T1D.

**Results:**

A total of 3818 children aged 2–16 years were recruited, with 14.2% (*n* = 542) having a “high genetic risk.” Of children with “high genetic risk” and without pre-existing T1D (*n* = 494), 7.0% (34/494) consented for autoantibody screening; 82.4% (28/34) who consented also completed the blood collection, and 7.1% (2/28) of them tested positive for multiple autoantibodies. Among children with pre-existing T1D (*n* = 91), 52% (*n* = 48) had a “high genetic risk.” In the sample of children with existing T1D, there was no relationship between genetic risk and age at T1D onset. A major factor in obtaining islet autoantibody testing was concern over SARS-CoV-2 exposure.

**Conclusions:**

Minimally invasive saliva sampling implemented using a genetic risk score can identify children at genetic risk of T1D. Consent for autoantibody screening, however, was limited largely due to the SARS-CoV-2 pandemic and need for blood collection.

**Supplementary Information:**

The online version contains supplementary material available at 10.1186/s13073-024-01305-8.

## Background

The genetic contribution to an individual’s risk of type 1 diabetes (T1D), based upon twin and family studies, is ~ 50% [1]. There is little “missing heritability” for T1D from large genome-wide association scan (GWAS) and fine mapping studies that have captured ~ 80–90% of the genetic risk [2–11]. However, the genetic contribution to disease initiation (development of islet autoimmunity) and disease progression (to clinical T1D) may not be the same as the variation recognized from comparison of cases with T1D versus controls. Genome-wide variation critical to the initiation of islet autoimmunity and progression from a single to multiple islet autoantibodies and to clinical T1D has not been interrogated widely in the human genome [12–14] and has focused previously on known disease-associated variants. In the longitudinal Diabetes AutoImmunity Study in the Young (DAISY) cohort, 160 islet autoantibody-positive participants (87 of whom progressed to clinical T1D) with whole genome sequencing identified four regions associated with progression, but none were “known” T1D susceptibility sites [15]. Thus, it is likely that novel genetic variation will need to be incorporated into models for better prediction of disease initiation, progression, and clinical diagnosis.

Genetic risk scores (GRS), composed of the summation of genotypes at each associated SNP, weighted by the odds ratio (effect size) of the SNP association with outcome, are now popular for predicting disease risk [16]. The GRS for T1D (T1D GRS), and for most common human diseases, have been based primarily on studies of European Caucasian ancestry, either from GWAS [2] or fine mapping [3], and used for prediction of risk and characteristics of autoimmunity [17–19]. An African-ancestry GRS (T1D_AFR_ GRS) had improved performance compared to the European GRS (T1D_EUR_ GRS) when tested on an independent series of African ancestry cases and controls [4], with T1D_AFR_ GRS AUC = 0.871 greater than T1D_EUR_ GRS AUC = 0.798 (*P* < 2.2 × 10^−16^).

The use of a T1D GRS to identify individuals at genetic risk at the population level has been motivated by the results of immune intervention in individuals with stage 1 (≥ two islet autoantibodies and normal glucose tolerance) or stage 2 (≥ two islet autoantibodies with dysglycemia) T1D to delay stage 3 (clinical disease) [20]. The Fr1da study was the first to assess a public health screening program in young children (median age 3.1 years) for islet autoantibodies [21]. Although the initial screening was based upon islet autoantibodies, a 46 SNP T1D GRS was not significantly associated with risk of progression from stage 1 to stage 2, or from stage 2 to stage 3, or risk of developing stage 3 T1D. In this report, we present results implemented in pediatric clinics within the Commonwealth of Virginia (USA) that included recruitment of a diverse population of children under the age of 16 years for genetic screening to identify those at “high genetic risk” for T1D, with follow-up for islet autoantibody screening among those at “high genetic risk.”

## Methods

In December 2016, the PrIMeD (Precision Individualized Medicine for Diabetes) program was established to form an interdisciplinary effort to investigate detection of those at risk of T1D using genetic screening and employment of the artificial pancreas to control T1D and develop a cure using immunologic methods of beta cell regeneration. The strategic goal of this report was to conduct a pilot study that could lead to establishment of a statewide network to provide genetic screening for T1D.

### Participant recruitment and baseline data collection

This study was approved by the University of Virginia Human Subjects Research Institutional Review Board, and a study ethicist was involved in the study design.

Children aged 2 to 16 years were offered saliva-based screening for genetic risk of T1D. Clinical research coordinators were stationed in the waiting rooms of pediatric and diabetes clinics that were selected for recruitment and study activities. Parent/guardians of children who were in clinics for well-child visits were approached for participation in the study and provided with a flyer describing the study with an educational section describing signs and symptoms of diabetes (Additional File 1: Fig S1) to increase awareness of the disease process in type 1 diabetes. Participation in the study was voluntary. Written informed consent/assent (Additional File 1: Document S1) was obtained on a tablet from the children’s parent/legal guardian in private space within each site.

A short baseline questionnaire (Additional File 1: Document S2) was obtained on a tablet to evaluate personal medical history and family history of health and diabetes, including contact information, family structure, self-reported ancestry, history of T1D and other autoimmune diseases, and if a family member had a prior diagnosis of diabetes (and evidence of hospitalization or diabetic ketoacidosis). The consent form and questionnaire were available in English and Spanish. The clinical research coordinator obtained a saliva sample (DNA Genotek Inc./Oragene•Discover|OGR-500) from each participant. Younger children, and those with difficulty producing enough saliva to spit, were offered an assisted collection that involved the clinical research coordinator using a small sponge-like swab to gently collect saliva from the participant’s inner cheek area. Unique barcodes were assigned to individuals to link consent forms, questionnaires, and saliva kits.

Clinical research coordinators hand-delivered the saliva specimens to the University of Virginia School of Medicine CAP/CLIA-certified Medical Genetics Laboratory where samples were logged and inventoried under CAP/CLIA protocols. DNA was extracted from the saliva samples using standard laboratory protocols, and batches were used as input to custom genotyping using a Fluidigm genotyping platform (BioMark HD Reader and Juno™ system). No saliva specimens were kept for long-term storage. The T1D-focused genotyping array was customized using 74 SNPs (Additional File 1: Table S1) to generate a T1D GRS [4, 17]. In T1D-associated regions, SNPs were selected using a stepwise conditional logistic regression model [3]. Lead variants independently associated with T1D were selected for the T1D-focused genotyping array. If the flanking sequence of a lead variant resulted in low-quality genotyping score, we replaced the variant with a proxy SNP in high linkage disequilibrium. Arrays were genotyped following the manufacturer’s (Fluidigm) protocol. The T1D GRS consists of 26 SNPs in the HLA region, three SNPs in *IFIH1* and *IL2RA*, two SNPs in *INS*, *DEXI*, *PTPN2*, and *TYK2*, and one SNP in the remaining T1D risk regions [3]. The genetic data produced by the custom array was subjected to both laboratory and statistical genetic quality control (e.g., SNP missingness and call rates).

### Genetic risk score (T1D GRS) classification

The genotyping panel produced raw data in batches of 96 samples that were analyzed using a KING [22] software script. Samples and SNPs that passed quality control (e.g., sample call rates ≥ 80%, SNP call rates ≥ 95%) were saved in a binary file, with the T1D GRS for each participant generated using PLINK [23] software. The T1D GRS was calculated by computing the sum of the T1D-associated risk alleles weighted by the effect size estimated from robust fine mapping data [3, 4]. The proposed threshold from the T1D GRS (≥ 5) corresponds to a modest sensitivity (0.446) with high specificity (0.955) as estimated from the T1DGC resource of 16,086 samples of European ancestry (6670 T1D cases). The prediction of T1D risk in the training set was high (AUC = 0.889) and represents a balance between missing fewer individuals at “true” high genetic risk versus being cost-effective (numbers requiring subsequent islet autoantibody testing).

Each participant was determined to be at “high genetic risk” or “not high genetic risk” based on a selected T1D GRS threshold (“high” defined by T1D GRS ≥ 5). In a previous test/validation case–control sample (data not published), this threshold provided ~ 85% sensitivity and ~ 80% specificity, roughly equating to a tenfold increased risk over population rate.

### Genetic risk communication

After application of the T1D GRS to define “high genetic risk” versus “not high genetic risk”, a report for each participant was generated. Since genetic risk was viewed as a research test, and was not CAP/CLIA certified, the information was not entered into the participant’s electronic health record. Results of the T1D GRS were returned to parents/legal guardians of the study participants, with approaches dependent upon disease status of the participant (with or without diagnosed T1D) and the T1D GRS value (“high” versus “not high”).

For participants with a “high genetic risk,” letters were mailed following either a successful phone contact (Additional File 1: Document S3) or 3 failed attempts at phone contact by a study pediatrician or genetic counselor. The letter provided basic facts about genetic and environmental risk factors for T1D, clinical signs and symptoms of hyperglycemia, and an interpretation of the T1D GRS. In addition, the letter included statements that genetic risk is “not destiny” (those at “high” genetic risk may not develop T1D; those at “not high” genetic risk may yet develop the disease).

For those participants at “not high genetic risk,” letters were mailed without initiating a phone call (Additional File 1: Document S4). All participants had access to a study phone line and email for questions. In addition, a certified genetic counselor was available to address questions from individuals that might impact their willingness to participate in the study as well as to provide additional risk interpretation for parents/guardians and participants after receipt of genetic risk results.

### Islet autoantibody testing

Parents/guardians of study participants eligible for islet autoantibody testing were identified, and a separate written informed consent/assent was obtained, blood collection (venipuncture) was scheduled, and specimens were shipped to the Clinical Immunology Laboratory at the Barbara Davis Center for Diabetes (BDC). The BDC measured islet autoantibodies to insulin (IAA), GAD65 (GADA), IA-2 (IA-2A), and ZnT8 (ZnT8A) using radio-binding assays [24]. In the 2020 IASP Workshop, sensitivity and specificity for IAA was 62% and 99%, for GADA 78% and 99%, for IA-2 72% and 100%, and for ZnT8 74% and 100%, respectively.

Islet autoantibody screening results were returned to PrIMeD using a secure HIPAA-compliant electronic portal. Parents/guardians of participants with either 0 or 1 autoantibodies detected received a mailed copy of their results with interpretation; results were considered “research” and not placed in the electronic health record. For those participants with no islet autoantibodies detected, repeat screening was recommended in 3 years; for those with one islet autoantibody detected, repeat screening was recommended in 1 year.

Clinically meaningful autoantibody screening results (≥ 2 islet autoantibodies detected) were reported to the parent/guardian by the PRiMeD pediatric endocrinologist, followed by a mailed copy of the results. A CLIA-certified report was generated and placed in the participant’s electronic health record, unless requested otherwise. For participants at “high genetic risk” with ≥ 2 islet autoantibodies, parents/guardians were presented with options for engagement in structured clinical monitoring, e.g., home fingerstick self-monitoring of blood glucose (SMBG), continuous glucose monitoring (CGM), or hemoglobin A1c (HbA1c) testing. Information on volunteering for participation in immune therapy trials (e.g., TrialNet) was provided.

### Follow-up of study participants

At least 1 year after study entry, a follow-up survey was conducted by clinical research coordinators. The survey was administered by telephone or email (for those with 3 failed phone contact attempts). Email attempts included an individualized link to the survey in Qualtrics HSD®, a HIPAA-compliant web-based survey platform. The follow-up questionnaire (Additional File 1: Document S5) obtained updated participant contact information and health history (T1D and other autoimmune disease development) of the participants and of their relatives.

### Statistical analyses

Statistical analyses using were performed R version 4.2.1, employing the *lm()* routine with the T1D GRS as the outcome variable and age as the predictor; covariates included sex, family history, and self-reported ancestry. In one secondary analysis, the T1D GRS was partitioned into those SNPs in the HLA region (in the human major histocompatibility complex, MHC) and those not in the MHC, to determine if source of genetic information (MHC versus not) had an association with age at onset. In a second analysis, the full T1D GRS was evaluated for association with early versus late age at onset (early ≤ 5 years). Significance was considered for *P* ≤ 0.05 in all analyses.

### Study implementation

Three sites affiliated with the University of Virginia Health System (UVA) were chosen to initiate recruitment—UVA Birdsong Clinic, UVA Pediatrics/Battle Building, Charlottesville, VA; Northridge Pediatrics, UVA Medical Park, Charlottesville, VA; Orange Pediatrics, Orange Medical Center, Orange, VA. These sites represent a university clinic environment (Birdsong), a private clinic environment (Northridge), and a rural clinic environment (Orange). Clinical research coordinators were hired in February 2018, one for each clinic site, and started a month of training for interaction with potential participants and their parent/legal guardian. A 2-week “shadowing” of each clinic was used to ascertain clinic-specific patterns of activities and to establish the optimal space for privacy during the consent/assent process. Staff were trained for collection of data and specimens in a manner to ensure minimum impact on clinic patient flow. Active recruitment was initiated May 2018. Ultimately, 12 clinical sites (university, private-practice, and diabetes subspecialty) from eight practices were used for recruitment into the study.

## Results

### Recruitment

Recruitment across all sites occurred from May 2018 until March 2021, with a target of 10,000 participants to be enrolled. A total of 7031 participants and their parents/legal guardians were approached; 61% agreed to participate and provided informed consent, and 90% of consented participants (*n* = 3818) completed the baseline study protocol and provided medical history data and a saliva sample. Descriptive statistics are provided in Table 1.

**Table 1:** Description of study participants (*N* = 3818)

Variable		Number (%)
Sex	Female	1859 (48.7%)
	Male	1959 (51.3%)
Age at recruitment (years)^a^	2–5	1143 (30.0%)
	6–9	1163 (30.4%)
	10–12	816 (21.4%)
	13–16	694 (18.2%)
Ancestry	White and LatinX	3163 (82.8%)
	Black	693 (18.2%)
	Asian	136 (3.6%)
	Other/not specified	84 (2.2%)
T1D at baseline (*n* = 3818)?	Yes, prevalent	91 (2.4%)
	No	3727 (97.6%)
	FDR with T1D^b^	208 (6.4%)
	No FDR with T1D	3064 (93.6%)
T1D at follow-up? (*n* = 2975)	Yes, incident	21 (0.7%)
	No	2954 (97.6%)
	FDR with T1D^b^	71 (2.4%)
	No FDR with T1D	2883 (97.6%)
Genetic risk score (high ≥ 5)	High	542 (14.2%)
	Not High	3276 (85.8%)

*T1D* type 1 diabetes, *FDR* first-degree relative

^a^Two participants were age 17 years (beyond the pre-selected age category) yet were enrolled in the study per IRB exception, given prior participation of a sibling; these participants (*n* = 2) are not included in the age distribution but are included in other demographic information

^b^Four hundred fifty-five participants did not provide data on having a first-degree relative (FDR) with type 1 diabetes (T1D) at baseline

Participants were approximately equally distributed by sex (51.3% [*n* = 1959] male) and captured a representative population in Virginia (82.8% white, 18.2% black or African American, 3.6% Asian-ancestry, and 2.2% other). In addition to those who entered the study, 12.8% of those approached were interested in participating but at a future time, and 26.6% refused. Four additional participants provided informed consent and later withdrew from the study (not included in the recruitment statistics) and data/samples destroyed. Recruitment was paused due to the SARS-CoV-2 (COVID-19) pandemic and restrictions related to hospital/clinic availability and reduced clinical and research operations. Only 317 participants were recruited and completed the protocol following re-opening of clinic sites.

### Screening yield

**Fig. 1:**
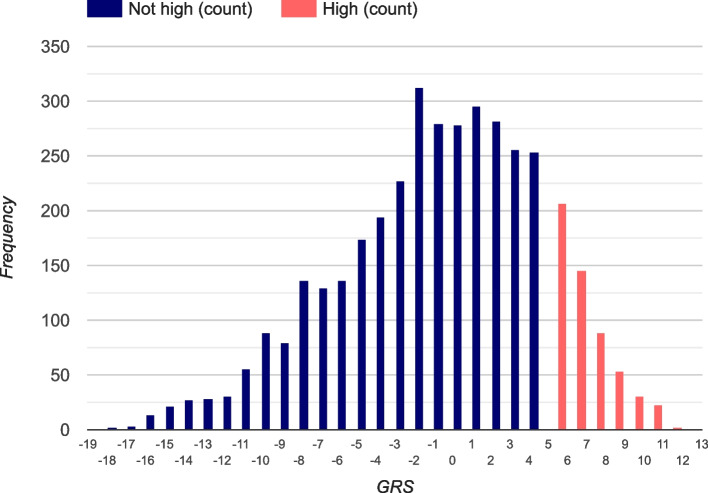
Frequency of study participants by T1D GRS: “not high” 3276 (85.8%), “high” 542 (14.2%)

Overall, 14.2% (542/3818) of participants were classified as having a “high genetic risk” of T1D (Fig. 1). Of those at “high genetic risk,” 91.1% (*n* = 494) were of self-reported white ancestry, 9.6% (*n* = 55) black or African American ancestry, 1.9% (*n* = 11) Asian ancestry, and 1.75% (*n* = 10) other/not specified ancestry.

### Genetic risk in those with type 1 diabetes

**Fig. 2:**
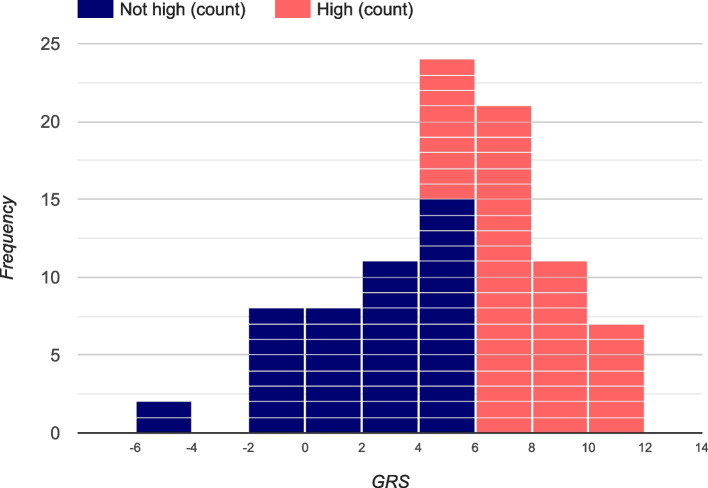
T1D GRS in participants with existing type 1 diabetes

Prevalent T1D was observed in 2.4% (*n* = 91) of those recruited; 52.2% (*n* = 48) had “high genetic risk” (Fig. 2), and 10 participants had a T1D GRS below the median. There was no significant difference in the mean (or median) age at ascertainment of participants by T1D GRS, with those at “high” genetic risk (mean: 9.4 + 4.1 years; median: 9.0 years) slightly older than those at “not high” genetic risk (mean: 8.9 + 4.0 years; median: 9.0 years). In participants with T1D, there was no significant association of T1D GRS with age at onset (*β* =  − 0.04 ± 0.09; *P* = 0.66); furthermore, there was no significant association of T1D GRS with age at onset after stratification by self-reported ancestry or early (by 5 years) age at onset. In the 48 participants with prevalent T1D and “high genetic risk,” only four had an HLA GRS that was below the threshold for “high genetic risk” (T1D GRS > 5.0); in these four cases, the non-HLA SNPs increased their GRS into the “high genetic risk” group. In the 43 participants with prevalent T1D but “not high genetic risk,” the largest HLA GRS = 4.848, yet the non-HLA SNPs had a negative GRS. Thus, the utility of including non-HLA SNPs is limited, and it could be argued that the 26 HLA SNPs in the panel would be sufficient to capture the majority of genetic risk of T1D.

### HLA contribution to type 1 diabetes risk

**Fig. 3:**
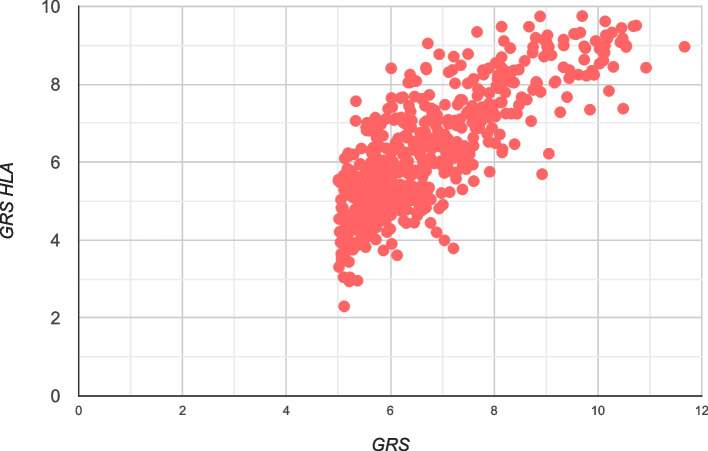
HLA and non-HLA impact in study participants with a high T1D GRS (*n* = 542)

Approximately 80% of participants classified with “high genetic risk” (T1D GRS ≥ 5) would also be classified as “high genetic risk” by restricting the T1D GRS to using only those SNPs in the MHC (Fig. 3). The T1D GRS due to HLA was not significantly associated with age at onset in those with T1D (*β*_HLA_ =  − 0.03 ± 0.09, *P* = 0.76). A similar relationship (*β*_nonHLA_ =  − 0.01 ± 0.03, *P* = 0.59) was observed when only considering non-HLA variants.

### Islet autoantibody screening

A total of 494 participants without T1D and at “high genetic risk” were eligible for islet autoantibody screening. Due to the SARS-CoV-2 pandemic, relatively few participated in collection of blood for islet autoantibody testing. Of 494 participants, only 34 consented, with 28 providing a sample for the islet autoantibody testing. Of those tested, 89.3% (*n* = 25) were negative for all islet autoantibodies and were recommended to repeat islet autoantibody testing in ~ 3 years. One participant was positive for one islet autoantibody (GADA) with a recommendation to repeat testing in ~ 1 year. Two participants tested positive for ≥ 2 islet autoantibodies (both participants were positive for GADA, IAA, and ZnT8A) and were recommended to provide the result to their primary care physician and offered detailed surveillance/monitoring with contact information for ongoing immune therapy trials.

### Follow-up of participants

A total of 2096 (55%) parents/legal guardians of participants completed the follow-up survey at least 1 year after study entry. Those not completing the follow-up survey were from loss to follow-up (*n* = 1017) or ineligibility for the survey (*n* = 753, primarily due to age beyond 18 years or less than 1 year of follow-up). 19.4% of participants who did not complete the follow-up survey were at “high genetic risk.” Two participants (of 2096, or 0.095%) reported development of T1D during the follow-up period; one participant was “high genetic risk” and one participant was “not high genetic risk.” One of these two participants had a first-degree relative with T1D.

## Discussion

Advances that motivate screening for genetic risk of T1D risk in the general population include (1) the knowledge of genetic variation associated with disease (e.g., [5, 6, 11]) and development of genetic risk scores in complex human diseases (e.g., [19, 25]); (2) prediction of risk using islet autoantibody screening [26, 27] and (3) the design of immune interventions [20, 28] and other agents [29, 30] focused on delaying/blocking onset of clinical (stage 3) disease in individuals at high risk. Early detection motivates intensive monitoring that could practically eliminate DKA at onset [31–33]. These advances suggest that genetics, coupled with autoantibody screening, could be tools in community/population screening.

To our knowledge, this is the first study that recruited children for genetic screening of T1D risk that included a broad age group (between 2 and 16 years), diverse ancestry, and urban/rural environments. In addition, recruitment included those with prevalent disease that permits estimation of genetic penetrance (risk of disease given a T1D GRS). In addition, a short-term follow-up survey was conducted to determine development of T1D or other autoimmune diseases. Among participants with T1D in our study, one half (*n* = 48) had a T1D GRS that was considered “high genetic risk,” consistent with the expectation that ~ 50% of T1D risk is due to genetic factors. Of the children with high genetic risk and no evidence of T1D, only 26 (5.7%) completed islet autoantibody testing, with two participants testing positive for multiple autoantibodies (stage 1). This low rate of testing was attributed, in large part, to the SARS-CoV-2 pandemic.

The current standard for islet autoantibody screening in T1D is the Fr1da study [21], a large, community-based study that utilized targeted (pre-school, 2–6 years of age) two-stage autoantibody detection process. The initial report on over 90,000 children screened demonstrated the feasibility of primary care physicians in a socialized healthcare environment collecting a capillary blood sample and testing for three islet autoantibodies (with repeat testing of a venous blood sample in those with two or more positive as well as a random blood glucose). In Fr1da, 280 children (0.31%) had two or more islet autoantibodies and were offered participation in an oral insulin intervention trial (NCT02620072), an extension of a previous, smaller trial [34] that demonstrated safety but no impact on immune response. Of those, 62 (22.1%) developed stage 3 (clinical) T1D. Through standard monitoring at timed follow-up examinations in Germany (at 2-to-6-month intervals), the risk of diabetic ketoacidosis was dramatically reduced (in two participants, 3.2% compared to 40–60% without monitoring).

The German health care system has extremely high compliance for childhood health-care visits, permitting standardized screening opportunities with a 10€ reimbursement for each sample collection. In contrast, PrIMeD deployed clinical research coordinators in clinic waiting rooms, approaching individuals with their children present predominantly for “healthy child” visits, a protocol with significantly increased cost (non-clinic personnel required) that was designed to avoid altering patient flow. In PrIMeD, there was high (over 60%), but not complete, agreement to participate in this study and the required venipuncture for islet autoantibody testing represented a barrier for many participants. The Fr1da design in its healthcare setting would be scalable, although unlikely applicable to many countries, including the USA.

The use of genetic information for detection of individuals at risk of disease has been growing through the development of GRS and, more recently, polygenic risk scores (PRS). The importance of transferability of any GRS to non-European ancestry populations has been highlighted in the UK Biobank [35], in which over 800 traits had PRS developed and evaluated by predicted performance and transferability in individuals of African and Asian ancestry. For T1D, the white UK Biobank British PRS models was highly significant (*P* = 3.8 × 10^−14^) with outstanding predictive performance in white British ancestry; however, the predictive performance was significantly worse when transferred to individuals of African or South Asian ancestry. These results emphasize the necessity of incorporating data from cohorts with diverse ancestry to establish useful scores for global detection of T1D genetic risk.

Estimates of the number of subjects expected to be detected with “high genetic risk” who ultimately develop stage 1 (multiple autoantibodies but normal glucose tolerance) T1D is uncertain due to few existing studies, differences in study designs, and lack of study populations of non-European ancestry. In TEDDY [36], a T1D GRS consisting of 41 SNPs found that at T1D GRS in the upper quartile increased the risk of developing multiple autoantibodies from 5.8% by age 6 years to 11.0% (compared to 4.1% in the low T1D GRS baseline risk). The risk of developing stage 3 (clinical) T1D by age 10 years increased from 3.7 to 7.6% in those with a high T1D GRS (compared to 2.7% in the comparison group). TEDDY participants with a T1D GRS in the lowest quartile had a slower progression from single to multiple autoantibodies, while those with the T1D GRS in the highest quartile had faster progression from single to multiple autoantibodies, progression from multiple autoantibodies to T1D, and an earlier age at islet autoantibody development [14]. In DAISY [37], a T1D GRS using 10 SNPs significantly predicted progression to diabetes, with inclusion of the T1D GRS with HLA out-performing HLA genotype risk prediction alone. In the TrialNet Pathways to Prevention participants, a T1D GRS with 30 SNPs replicated the increased rate of progression to T1D as well as the higher T1D GRS associated with increased progression rate from single to multiple autoantibodies [38]. Together, these studies suggest the T1D GRS contains variants specific to the development and presence of islet autoantibodies, rapid progression to multiple autoantibodies, and development of T1D.

The cost of implementing PrIMeD was significant, with high personnel cost of clinical research coordinators staffing individual clinic sites. There is evidence, however, that investment in T1D screening can be cost effective. It has been estimated that $4700 was required per case of T1D detected for children and adolescents enrolled in a screening program, with $14,000 per case detected for routine screening in Denver, CO [39]. The 20% reduction in DKA events combined with 0.1% improvement in HbA1c levels would be needed for the program to reach a value threshold of $50,000–$150,000 per quality-adjusted life-year. The Denver clinic site (BDC) is an established care center for T1D and in a major metropolitan area. In other areas, however, the infrastructure cost to establish a screening program may be prohibitive. Although the current estimate of genetic ($5/person with the African-ancestry HLA SNP panel) and islet autoantibody surveillance ($10/test with new in-home kits) is scalable, the cost still needs to approach the projected $1/test for HLA and $0.03/test for islet autoantibodies estimated for a favorable cost–benefit [40].

In the PrIMeD, venipuncture for islet autoantibody determination was viewed as a barrier due to its invasiveness, suggesting that other (minimally invasive) methods of sample collection should be evaluated. In contrast to genetic risk screening in which the collection of the saliva sample was non-invasive, familiar to parents/guardians, and performed while waiting for a scheduled appointment, the autoantibody component required a separate, scheduled blood draw in a clinical laboratory setting. Scheduling an additional visit for phlebotomy, especially when it is for screening and not actual medical care, was challenging and represented a significant barrier. At the time of this study, there were few alternatives to the in-clinic blood collection for islet autoantibody testing; however, newer technology now makes in-home testing (with postal return) a viable and cost-effective (~ $10/test) option. These newer kits can be provided at a regularly scheduled clinic visit (by a health care provider) or through direct mailing, thereby likely increasing the proportion of participants willing to have islet autoantibody testing and evaluation for staging of type 1 diabetes.

It should be recognized, however, that the PrIMeD study has important limitations. First, the sample size was less than projected, in large part due to the inability to recruit during the SARS-CoV-2 pandemic. Second, the number of “high genetic risk” participants with islet autoantibody screening was limited due, in large part, to the SARS-CoV-2 pandemic and the need for venipuncture; however, use of a saliva sampling approach to obtain a T1D GRS was successfully implemented in multiple clinic settings. These results suggest that genetic screening is accepted by the community, although barriers to subsequent autoantibody testing using venipuncture remain. Potential barriers for implementation, including issues of scaling and cost, could be reduced by developing an in-home screening program (available now for saliva collection and mailing to a central laboratory) for autoantibody testing. Additional research combining a T1D GRS and autoantibody monitoring should consider utilizing existing infrastructure to reduce costs and identify more acceptable methods of sample collection for autoantibody monitoring and recruitment to increase diversity from other regions of the USA and globally. Future research into the determination of knowledge of type 1 diabetes pre- and post-screening in those with either “high” or “not high” genetic risk is needed in order to understand the long-term perception of risk.

## Conclusions

Early detection of type 1 diabetes becomes increasingly important with the development of immune interventions (e.g., teplizumab with FDA approval in stage 2 disease). Our study suggests that a T1D GRS approach is implementable in the USA at a population level, based upon acceptance of saliva sampling as a non-invasive application. Future use of kits for in-home saliva collection for DNA/genotyping and minimal blood for islet autoantibody testing with distribution by health care staff, school staff, or public health service with postal self-return will likely enhance adherence to screening guidelines. Furthermore, the use of blood spots has been shown to provide sufficient DNA for determination of genetic risk scores. As the primary purpose of screening is detection of those individuals at risk, the use of a T1D GRS is one component of the screening process, the other being islet autoantibody testing. The combination of genetic screening (that is performed once) and islet autoantibody screening (that may require testing over a “risk period”) will be necessary to precisely “stage” individuals for risk of type 1 diabetes.

Enrollment for genetic screening for risk of T1D in our diverse community medical setting (primarily pediatric waiting rooms) was a success, with over two thirds of those approached willing to participate immediately, with another ~ 15% willing to participate at later date. There were ~ 14% of participants classified as having a “high genetic risk” of T1D, in contrast to ~ 50% of those with existing T1D. This study demonstrates general acceptance of genetic testing across diverse community settings and provides evidence that the T1D GRS may be implemented and accepted on a large scale. The cost-effectiveness aspect, while not addressed here, is dependent upon cost of tests and avoidance of the serious complication of undetected progression to stage 3 (clinical) type 1 diabetes and diabetic ketoacidosis that requires hospitalization, increased difficulty in glucose control, and increased risk of diabetic complications.

### Supplementary Information

**Additional file 1: Document S1.** Study consent form. The consent form used for parent/guardian assent of minors. **Document S2.** Baseline questionnaire. Information obtained on the participant and family members from the parent/guardian at time of enrollment. **Document S3.** “High Genetic Risk” letter. For those participants with a high genetic risk score for type 1 diabetes, the letter sent to the parent/guardian of the participant in addition to contact by telephone (or only information if not contacted after three attempts). **Document S4.** “Not High Genetic Risk” Letter. For those participants that do not have a high genetic risk score, the letter sent to the parent/guardian. **Document S5.** Follow-up questionnaire. The form used for clinical research coordinators to obtain information through telephone contact with parent/guardian of participant to update personal and family health status. **Fig S1.** Study flyer. Information provided at each recruitment site for distribution to parent/guardians concerning the study and signs/symptoms of type 1 diabetes. **Table S1.** T1D genetic risk score SNP list. List of SNPs (rsID), the effect allele, the frequency of the effect allele, the weight (effect size; positive numbers are extent of “risk” and negative numbers are “protection”), the chromosome designation of the SNP, the position of the SNP on the chromosome, and the alternative (non-effect) allele.

## Data Availability

Summary statistics for creation of the T1D GRS are publicly available in the NIH database of Genotypes and Phenotypes (dbGaP) with accession numbers phs000911.v1.p1 (https://www.ncbi.nlm.nih.gov/projects/gap/cgi-bin/study.cgi?study_id=phs000911.v1.p1) and phs002468.v1.p1 (https://www.ncbi.nlm.nih.gov/projects/gap/cgi-bin/study.cgi?study_id=phs002468.v1.p1). SNP effect size estimates for the T1D GRS can also be obtained from the PGS Catalog (https://www.pgscatalog.org/) under PGS ID: PGS000021 (https://www.pgscatalog.org/score/PGS000021/) and PGS000023 (https://www.pgscatalog.org/score/PGS000023/). Access to the coded individual-level data used in this manuscript is deposited into the Accelerating Medicines Partnership Common Metabolic Diseases (AMP CMD, https://t1d.hugeamp.org/) Knowledge Portal.

## Abbreviations

DKA: Diabetic ketoacidosis
GRS: Genetic risk score
PrIMeD: Precision Individualized Medicine for Diabetes
T1D: Type 1 diabetes
