# Supplementary material for: Implementation of type 1 diabetes genetic risk screening in children in diverse communities: the Virginia PrIMeD project
Source: Genome Med. 2024 Feb 14;16:31. doi: 10.1186/s13073-024-01305-8 (PMC10865687; doi:10.1186/s13073-024-01305-8)
Supplement: Supplementary file 1 — Additional file 1: Document S1. Study consent form. The consent form used for parent/guardian assent of minors. Document S2. Baseline questionnaire. Information obtained on the participant and family members from the parent/guardian at time of enrollment. Document S3. “High Genetic Risk” letter. For those participants with a high genetic risk score for type 1 diabetes, the letter sent to the parent/guardian of the participant in addition to contact by telephone (or only information if not contacted after three attempts). Document S4. “Not High Genetic Risk” Letter. For those participants that do not have a high genetic risk score, the letter sent to the parent/guardian. Document S5. Follow-up questionnaire. The form used for clinical research coordinators to obtain information through telephone contact with parent/guardian of participant to update personal and family health status. Fig S1. Study flyer. Information provided at each recruitment site for distribution to parent/guardians concerning the study and signs/symptoms of type 1 diabetes. Table S1. T1D genetic risk score SNP list. List of SNPs (rsID), the effect allele, the frequency of the effect allele, the weight (effect size; positive numbers are extent of “risk” and negative numbers are “protection”), the chromosome designation of the SNP, the position of the SNP on the chromosome, and the alternative (non-effect) allele. [file 13073_2024_1305_MOESM1_ESM.pdf]

Document S1. Study consent form.

Collection of Samples and Health Information for Genetic Research

Parents' or Guardians' Permission for Your Child to Be in a Research Study

Agreement of a Child to Be in a Research Study Age 15 to <18

In this form "you" means the child in the study *and* the parent or guardian.

- ✓ If you are the parent or guardian, you are being asked to give permission for your child to be in this study.
- ✓ If you are the child, you are being asked if you agree to be in this study.

In this form "we" means the researchers and staff involved in running this study at the University of Virginia.

Participant's Name_______________________________ **Medical Record #** ___________________

**Principal Investigator:**	**Dr. Stephen S. Rich, PhD**<br>University of Virginia<br>Center for Public Health Genomics<br>P.O. Box 800717<br>Telephone: (434) 982-3228 Email: ssr4n@virginia.edu
**Sponsor:**	University of Virginia Strategic Investment Fund

What is the Purpose of this Form?

This form will help you decide if you want to be in the research study. You need to be informed about the study before you can decide if you want to be in it. You should have all your questions answered before you give your permission, or consent, to be in the study. This is called an "informed consent" form because it informs you before you sign to give your consent. We encourage you to discuss this with your family and friends.

If you do want to be in the study, you will need to sign this form to give your consent. You will get your own copy of this signed form for your records.

You are being asked to participate in the Virginia PrIMeD (Precision Individualized Medicine for Diabetes) Project: Genetic Risk of Type 1 Diabetes. This is an effort to identify individuals at high genetic risk of type 1 (or juvenile) diabetes.

Why is this research being done?

Type 1 (or juvenile) diabetes is an important health problem that affects many people, and the majority (over 90%) of those who have type 1 diabetes do not have a family member with the disease. Doctors know that genes (DNA) play an important role in type 1 diabetes; about half of the risk of type 1 diabetes is due to genetics. While not all children with a family history (or high genetic risk) of diabetes will eventually develop the disease, identifying those at highest risk may allow for closer monitoring for possible development of type 1 diabetes by your clinician over time. We hope that this will improve health outcomes by reducing potential complications

related to initial uncontrolled blood sugar and unrecognized start of the disease.
Children with undiagnosed type 1 diabetes often do not appear sick until their uncontrolled blood sugars are so high that they experience a critical event, called diabetic ketoacidosis, which results in hospitalization. Diabetic ketoacidosis often presents rapidly and is a serious, potentially life-threatening condition that may result in loss of consciousness and organ damage. It is often during this hospitalization event that a child is first diagnosed with type 1 diabetes.

Recent scientific work has given us a better understanding of which genetic factors are important predictors of type 1 diabetes genetic risk in children. The goal of the study is to identify those children who are at high genetic risk of type 1 diabetes based on these genetic markers that we measure, using our genetic risk calculator. It is important to realize that this study will not provide clinical diagnoses of type 1 diabetes, and that there are no clinical prevention measures that can be offered at this time. However, identification of children at high genetic risk provides valuable information for you and your clinician. You will become more alert for signs and symptoms of early onset of disease, and will potentially avoid the complications that often accompany the sudden clinical development of type 1 diabetes by avoiding diabetic ketoacidosis.

All procedures described within this consent form will be done for research purposes only, and we will not provide any clinical care.

What Sort of Research Will Be Done On Your Sample(s)?
You are being asked to provide a saliva (spit) specimen to be used for research. We will do genetic research on the DNA obtained from your saliva specimen to determine your genetic risk of type 1 diabetes.

Along with this specimen, researchers wish to collect some health information about you. Combining information from the specimen with information from your health records may be useful for this research. For this research, the following types of information could be included: family history of diabetes, medical events, or conditions related to diabetes.

No clinical care will be provided as part of this study. The purpose of these tests is not to diagnose any disease or abnormality you may have. Your clinician will report whether or not you are at high genetic risk of type 1 diabetes based on the DNA obtained from the saliva specimen and our genetic risk score formula. However, this test cannot determine whether or not you will ultimately develop type 1 diabetes. We will collect your contact information (including name, postal address, e-mail address, and telephone numbers) as part of this study, and will use this information to send you a copy of your genetic risk score results (high genetic risk or not high genetic risk for type 1 diabetes). These results will be sent within 8 weeks from the date we collect your specimen at the clinic. Your clinician may also contact you directly to discuss the results and to provide a more detailed interpretation.

We plan to do genetic research on the DNA in your saliva specimen. DNA is the material that makes up your genes. All living things are made of cells. Genes are the part of cells that contain the instructions that tell our bodies how to grow and work, and determine physical characteristics such as hair and eye color. Genes are passed from parent to child. Based on scientific research, we have developed a risk formula that will use the

genetic information from the saliva specimen to determine your genetic risk for type 1 diabetes. Only genetic factors related to type 1 diabetes risk will be measured in your specimen.

When these tests are completed, any leftover saliva or DNA sample will be discarded. This means there is no information that could be used by anyone to determine who provided the sample.

How Many People Will Take Part in This Study?

Our goal is to enroll at least 60,000 participants in this study over at least three years.

What will you have to do to give samples for research?

Collecting your saliva specimen will take place during your visit today after we obtain your written consent to participate in the study. The collection of your saliva specimen will take about 5-10 minutes, with a total of 30-45 minutes for participation in this study visit (including the consent process).

If you agree to be in this study, you will sign this consent form before any study related procedures take place.

<u>**Study Visit 1 (will take approximately 30-45 minutes)**</u>

- If you agree to be part of this study, you will be asked to complete a brief questionnaire regarding your medical history and your family history of diabetes.
- At the same visit, we will collect a saliva sample from your mouth to obtain DNA for determination of genetic risk.
    - <u>If you are under 5 years old,</u> a sterile swab (a sponge-like material on the end of a short stick) will be placed against the inside your cheek to collect saliva. The swab will then be placed into a collection tube in order to transfer the saliva into the tube. This will be repeated until we have obtained the amount of saliva needed for the study, as marked on the tube. This process is a safe and non-invasive procedure that takes about 5 minutes. This assisted saliva collection can also be used for those participants who have difficulty in using the unassisted collection kit.
    - <u>If you are 5 years of age or older,</u> we will use an unassisted kit to collect your saliva. We will give you a tube and ask you to spit saliva into it until the amount of saliva needed for the study is obtained, as marked on the tube. This is a safe and non-invasive procedure that takes about 10 minutes.

<u>**FOLLOW UP:**</u>

<u>**For all participants:**</u>

Your clinician will contact you to report whether or not you have a high genetic risk of type 1 diabetes based on the DNA results and our genetic risk score formula. Your clinician will provide detailed guidance on the interpretation of the genetic risk score for type 1 diabetes, and you should contact them with any questions you may have during the study. If you need additional information and counseling after speaking with your clinician, the study team can refer you to a genetic counselor.

Study staff will contact you once a year to conduct a short questionnaire to update your medical history for potential development of type 1 diabetes and related data.

How Will Your Sample(s) Be Labeled?
Your sample(s) will not be labeled with your name or other information that would identify you directly. Instead, it will have a unique code that allows for it to be linked to some of your health information. This link means that your specimen can be identified but only indirectly. We will only keep your specimen until the DNA is analyzed, at which point the specimen will be destroyed.

Which researchers can use your samples and what information about you can they have?
Your information and results of your genetic risk score may be shared with researchers at the University of Virginia and at other institutions or with the sponsor (if applicable). Dr. Rich will not give your name to other researchers who want to use your sample, but will only give them information like your age and what disease/condition you have. Those who would see the information would include researchers and the others listed under "Who will see your private information?" section of this consent document.

Some of the people who receive your information may share or release your information because they do not have to follow the privacy laws.

What Are the Benefits To Donating Your Sample(s) For Genetic Research?
Determining whether you are at high genetic risk for type 1 diabetes provides valuable information for you and your clinician, will allow for possible further monitoring by your clinician over time (separate from this study), and may avoid the complications that often accompany undiagnosed type 1 diabetes.

Will You Find Out the Results of the Research on Your Sample(s) for Genetic Research?
If you participate in this study, we will provide you with information about whether you are at high genetic risk of type 1 diabetes. If you do not want to receive your genetic risk information, you should not participate in the study.

If you do decide to participate in the study and receive your results the following information is important for you to know:
The specific DNA results of your genetic tests will not be disclosed to you, your health care provider, nor anyone in your family. However, your clinician will be informed regarding your genetic risk of type 1 diabetes based on the DNA we measured from their saliva specimen and our genetic risk score formula. No information about your participation in this study of genetic risk screening will be put into your medical record or shared, such as the actual genetic data (genetic sequences), the genetic risk score, or the consent form for participating in this study.

It is important to note that the genetic risk score for type 1 diabetes is based on research. It is not considered medical information or clinically actionable. We are not providing diagnoses of diabetes, so no clinical care or treatment are provided by participating in this study.

What Are The Risks of Donating Your Sample(s) For This Study?

Risks to Privacy from Genetic Research:
The main risk of allowing us to collect and use your samples and obtain limited health information for research is the potential loss of privacy. One of the risks to you is the release of information from your health information that you give us through answering our limited questions. We will not collect any information directly from your health records. The University of Virginia will do its best to protect your information so that facts about you and your health will be kept private. The chance that information identifying you will be given to someone else is very small. However, we cannot *guarantee* it will be safe. To further safeguard your privacy, information obtained from this research will not be placed in your medical record.
There are certain risks of having any health information given to other people by mistake. In the unlikely event that this happens, it could cause discrimination or mental harm to you or your family members if others were to see this information. The results of this study are not considered information that could discriminate you from getting or keeping certain kinds of insurance. Knowledge about genetic risk of type 1 diabetes could, however, hurt family relationships or increase anxiety during the years of risk.

We are only measuring a limited amount of your genetic data based on the saliva specimen and are not measuring your entire DNA sequence (your genome). Also, we are measuring genetic information that has no direct clinical importance, only that taken together, the genetic risk score accounts for the majority of risk for type 1 diabetes. Therefore, this study has only minor risks associated with it. For example, it is very unlikely that someone could identify you through your DNA even if they have another sample of your DNA.

Information about your complete genetic make-up could mean that you and your family members could face problems that could lead to getting or keeping some kinds of insurance or affect your ability to get or keep a job. This is based upon knowledge of specific genes that lead to disease. In this research, the number of genetic factors is small (96) and have no direct relationship to a genetic disease that may affect insurance or employment. To further minimize this risk, the results of these tests will not be given to anyone outside of the study staff and the results will not be put into your medical record. There is no way to predict all the possible risks of this research.

The saliva specimen is being collected for research purposes only; the saliva specimen is non-invasive and quick.

Risks of having your saliva specimen collected for DNA:
Risks and side effects related to the *assisted* collection of saliva specimen (for children <5 or those who have difficulty with the unassisted collection) for DNA include minor irritation of the cheek, which is unlikely.

- ✓ Minor discomfort (rare)

If we determine that you have a high genetic risk based on the measured DNA and our genetic risk score formula, it does not mean that you will develop type 1 diabetes, and there are no clinically actionable steps that can be taken related to the genetic risk score. The genetic risk result (high risk or not) is not medical information and

will not affect your medical care. No treatment will be provided as part of this study. Therefore, risks related to obtaining your genetic material are limited.

There is no way to predict all the possible risks of this research. However, one possible risk is psychological stress that may occur if you are found to be at high genetic risk for type 1 diabetes.

What If You Change Your Mind About Donating Your Sample(s) for Genetic Research?
Your specimen will only exist for a short time until the DNA is analyzed, and then the specimen will be destroyed. If you change your mind about letting us use your sample for genetic research and the specimen has not already been analyzed and destroyed, you will need to write to the Principal Investigator listed on the first page of this form to request that you sample be destroyed. We will then destroy any of your sample that has not already been used. If your sample has been used in genetic research, and you request to withdraw from the study, the information that we have learned will be removed as well.

What are your other choices if you do not join this study?
If you do not want to donate your sample for genetic research, your only choice is not to be in this study.
If you are a patient at UVa your usual care will not be affected if you decide not to participate in this study.
If you are an employee of UVa your job will not be affected if you decide not to participate in this study.
If you are a student at UVa, your grades will not be affected if you decide not to participate in this study.

Will You Be Paid For Donating Your Sample(s) for Genetic Research?

You ***will not*** be paid to donate your sample(s) for genetic research.

Will Donating Your Sample(s) Cost You Any Money?

There is no cost to you to have your samples collected or used for genetic research.

What if you are hurt in this study?
If you are hurt as a result of being in this study, there are no plans to pay you for medical expenses, lost wages, disability, or discomfort. The charges for any medical treatment you receive will be billed to your insurance. You will be responsible for any amount your insurance does not cover. You do not give up any legal rights, such as seeking compensation for injury, by signing this form.

How will your personal information be shared?
The UVa researchers are asking for your permission to gather, use and share information about you for this study. If you decide not to give your permission, you cannot be in this study, but you can continue to receive regular medical care at UVA.

If you sign this form, we may collect any or all of the following information about you:
- Personal information such as name, address, date of birth

- Your health information. This will include questionnaire data collected today and throughout follow-up. This may include a review of your medical records and test results from any of your doctors or health care providers
- Saliva specimen sample for genetic testing

Who will see your private information?

- The researchers to make sure they observe the effects of the study and understand its results
- People or committees that oversee the study to make sure it is conducted correctly
- People who evaluate study results

The information collected from you might be published in a medical journal. This would be done in a way that protects your privacy. No one will be able to find out from the article that you were in the study.

What if you sign the form but then decide you don't want your private information shared?

You can change your mind at any time. Your permission does not end unless you cancel it. To cancel it, please send a letter to the researchers listed on this form. Then you will no longer be in the study. No more information about you will be added to the database after your cancellation. The researchers will remove information about you that was collected before you ended your participation. UVa researchers will do everything possible to protect your privacy. However, they will need to share your information with people who may not have to follow the rules described above. Some of those people may be allowed to share/release your information without your permission.

Please contact the person listed below to:

- Learn more about the study
- Ask about the way the study is done or about treatments
- Report an illness, a research related injury, or other problem (you may also need to tell your regular doctors)
- Leave the study before it is finished
- Report a concern about the study

Stephen Rich, PhD
University of Virginia
Center for Public Health Genomics
P.O. Box 800717
Telephone: (434) 982-3228

What if you have a concern about a study?

You may also report a concern about a study or ask questions about your rights as a research subject by contacting the Institutional Review Board listed below.
University of Virginia Institutional Review Board for Health Sciences Research
PO Box 800483
Charlottesville, Virginia 22908 Telephone: 434-924-2620

When you call or write about a concern, please give as much information as you can. Include the name of the study leader, the IRB-HSR Number (at the top of this form), and details about the problem. This will help officials look into your concern. When reporting a concern, you do not have to give your name.

We have asked the federal government to issue a Certificate of Confidentiality, to help protect the privacy of your study records. If we receive a subpoena or court order demanding information from the study records that would identify you, we will use the Certificate to resist the demand. However, UVa will not use it in the following cases.

- You have agreed in writing to allow UVa to share the information with your employer, your insurance company for billing purposes, or someone else
- Reports to authorities where there is a danger that you may harm yourself or others, or if there is evidence of probable child or elder abuse or neglect.

In addition, the Certificate does not prevent government authorities who oversee research from reviewing this study. This Certificate does not mean that the government either approves or disapproves of this study. It just helps protect your privacy

In order to participate in this study, you must agree for collection of your saliva specimen for genetic research. If you do not want to allow for your saliva specimen to be used for genetic research, you should not consent to participate in this study. No matter what you decide to do, your decision will not affect your medical care. You can tell us your choice by checking one of the options below:

FUTURE CONTACT FOR OTHER RESEARCH STUDIES: In addition to the study activities related to the saliva specimen collection, DNA measurement, and calculation of a genetic risk score for type 1 diabetes, we would like to ask you about your consent for future potential research activities. If you agree to be contacted by researchers about potential future research studies related to diabetes, its complications, and other autoimmune diseases, please check "yes" below. This will not affect your participation in the study of genetic risk of type 1 diabetes.

I agree to be contacted in the future for potential participation in other research studies.
(Please check one box):

Yes ☐

No ☐

What does your signature mean?

Before you sign this form, please ask questions about any part of this study that is not clear to you. When you sign below, you are saying you understand the information we gave you about the study and in this form. You agree that your sample and questionnaire data may be used for genetic research as outlined in this consent.

Assent from Child (15-17 years of age)

Consent from the parent/guardian MUST be obtained before approaching the child for their assent.

___________________________	_________________________	_______
PARTICIPANT (SIGNATURE)	PARTICIPANT (PRINT)	DATE

To be completed for any child age 15 or above.

Person Obtaining Assent of the Child

Consent from the parent/guardian MUST be obtained before approaching the child for their assent.

By signing below you confirm that the study has been explained to the child (less than 18 years of age), all questions have been answered and the child has voluntarily agreed to participate.

___________________________	_________________________	_______
PERSON OBTAINING ASSENT (SIGNATURE)	PERSON OBTAINING ASSENT (PRINT)	DATE

Parental/ Guardian Permission

By signing below you confirm you have the legal authority to sign for this child.

___________________________	_________________________	______
PARENT/GUARDIAN (SIGNATURE)	PARENT/GUARDIAN (PRINT NAME)	DATE

Person Obtaining Parental/Guardian Permission

By signing below you confirm that you have fully explained this study to the parent/guardian, allowed them time to read the consent or have the consent read to them, and have answered all their questions.

________________________________	______________________________	________
PERSON OBTAINING PARENTAL/ GUARDIAN PERMISSION (SIGNATURE)	PERSON OBTAINING PARENTAL/GUARDIAN PERMISSION (PRINT NAME)	DATE

<u>**Consent from Impartial Witness**</u>

If this consent form is read to the subject because the subject is blind or illiterate, an impartial witness not affiliated with the research or study doctor must be present for the consenting process and sign the following statement. The subject may place an X on the Participant Signature line above.

I agree the information in this informed consent form was presented orally in my presence to the **identified individual(s)** who has had the opportunity to ask any questions he/she had about the study. I also agree that the **identified individual(s)** freely gave their informed consent to participate in this trial.

Please indicate with check box the identified individual(s):

☐ Minor Subject

☐ Parent(s)/Guardian of the subject

___________________________________	_________________________________	_________
IMPARTIAL WITNESS (SIGNATURE)	IMPARTIAL WITNESS (PRINT)	DATE

Document S2. Baseline questionnaire.

BASELINE QUESTIONNAIRE

Consent and Assent

**This box will be completed by the study staff member who will consent the participant.**
Name of consenter _______________________(First, middle, last)
Today's date ______ ________ ___________<br>MM DD YYYY

[Medical Record Number, Clinic, Study Staff member involved, date of questionnaire should have been entered previously or automatically generated based on the information entered into the consent forms]

Participant's (child) full name: __________________________(First, middle, last)

Full name of parent/guardian completing this form:

____(First, middle, last)

Age of child: ___________ (hand-enter #, must be between 0 and 16)

- *If age <7, prompt completion of consent form and require parent/guardian signature*
- *If age 7-14, prompt completion of consent form and require signatures from parent/guardian and child.*
- *If age >14, prompt completion of consent form with required parent/guardian signature, and require completion of minor assent form with required minor signature.*

Consent and assent forms will include parent/guardian email address*, and consent form will be automatically emailed to parent/guardian upon completion. If parent/guardian does not have an email address, clinician will create a pdf and print.*

If the appropriate consent and assent are completed, system will prompt to complete the following information:

Participant Contact Information

We would like to collect contact information for you and your child in order to share study information with you and, with your permission, to follow your child over time for future health outcomes related to type 1 diabetes.

Full name of parent/guardian completing this form:

____(First, middle, last)

Full name of other parent/guardian:

____(First, middle, last)

Primary caretaker phone number

- Home/cell (XXX) XXX-XXXX
- Work (XXX) XXX-XXXX

Preferred phone contact: ☐ home/cell ☐ work

Primary caretaker mailing address (Street, Apt/PO Box, City, State, Zip Code)

____

Primary caretaker email address:

____ (force to have @ sign)

Note: This email address will be used to automatically email you a copy of the consent form

Demographics and Health History

Now we'd like to ask some questions **about your child** who is participating in this study.

Child's date of Birth ______ ________ ___________
MM DD YYYY
(must allow min age of child to be 2 and max age of child to be <19)

Are any of the child's siblings or other household members involved in this study?
☐ Yes ☐ No

Sex ☐ Male ☐ Female

Child's race (check all that apply)
☐ White
☐ Black or African American
☐ Asian
☐ Native Hawaiian or other Pacific Islander
☐ Native American, Alaskan Native, Aboriginal Canadian, Aboriginal ☐Australian
☐ Don't know

Ethnicity: Is your child of Hispanic, Latino, or Spanish origin?
☐ Yes ☐ No ☐ Don't know

Does the child have a <u>family history</u> of Type 1 diabetes? Please base your answer on blood relatives.
☐ Yes [If Yes, prompted to answer next question.]
☐ No
☐ Don't know

[This question is only prompted by people who answer "Yes" above to family history of diabetes]
Which of your child's family members has been diagnosed with type 1 diabetes? Please only respond based on blood relatives.
☐ mother ☐ father ☐ sibling ☐ other

Does the child have a <u>family history</u> of autoimmune disease, other than Type 1 diabetes? Please base your answer on blood relatives.
☐ Yes [If Yes, prompted to answer next question.]
☐ No
☐ Don't know

If yes, please mark all autoimmune conditions diagnosed among your child's blood relatives:
☐ Thyroid (Hashimoto and/or Graves)
☐ Celiac Disease

☐ Addison Disease
☐ Other (please list ________________________)
☐ Blood relative has been diagnosed with autoimmune disease, but I don't know which condition

Has your medical provider ever told you that your child had high blood sugar?
☐ Yes ☐ No ☐ Don't know

Has your child ever been diagnosed with diabetes?
☐ Yes ☐ No ☐ Don't know

[If yes to above question about diabetes, prompt to answer the next four questions]
At what age was your child diagnosed with diabetes?
_______________ (fill in, require # between 0-18)

Is your child currently on any medication for diabetes?
☐ Yes ☐ No ☐ Don't know

Has your child ever been hospitalized for diabetes?
☐ Yes ☐ No ☐ Don't know
[If yes, fill in month and year]

Has your child ever had diabetic ketoacidosis (DKA)?
☐ Yes ☐ No ☐ Don't know

Has your child ever been diagnosed with an autoimmune disease, other than Type 1 diabetes?
☐ Yes ☐ No ☐ Don't know

[If yes to above question about autoimmune disease, prompt to answer the next question]
If yes, please mark all autoimmune conditions:
☐ Thyroid (Hashimoto and/or Graves)
☐ Celiac Disease
☐ Addison Disease
☐ Other (please list ________________________)
☐ Child has been diagnosed with autoimmune disease, but I don't know which condition

Thank you for participating in The Virginia PrIMeD (Precision Individualized Medicine for Diabetes) Project: Genetic Risk of Type 1 Diabetes!

Document S3. "High Genetic Risk" letter.

University of Virginia School of Medicine
Center for Public Health Genomics

University of Virginia Children's Hospital

PrIMeD
Precision Individualized Medicine for Diabetes

October 23, 2018

Recipient Name
Address line 1
City, State/Province and Postal Code

Dear parent/guardian of [NAME],

Thank you for participating in the research study, IRB-HSR#20939: The Virginia PrIMeD (Precision Individualized Medicine for Diabetes) Project: Genetic Risk of Type 1 Diabetes. We have evaluated the saliva specimen that [NAME] provided during the study visit, and have performed the DNA test. Based upon current knowledge of the genetic contribution to risk of type 1 diabetes, we believe that [NAME] ***is*** considered to be at high genetic risk.

You have been contacted by a member of our team (either a pediatrician from your primary care provider or our study pediatrician at UVA, Dr. {Staff NAME}) to discuss the risk results for [NAME]. In addition to the materials found here, you have been provided with information about the genetics of type 1 diabetes in relation to this study.

What does this result mean?
We define high genetic risk for type 1 diabetes as a ten-fold greater risk compared to the general population. We understand that your child has been diagnosed with type 1 diabetes. Among children with a high genetic risk result in this study, we expect that 4 out of 100 children will develop the disease sometime during their lifetime.

This test was done for research purposes and is not based on an FDA-approved clinical test, however you may choose to bring this letter to your next primary care provider visit and share this information with them.

We thank you for participating in the study. Please contact us at (434) 982-0507 with any questions or concerns.

Sincerely,

{Staff NAME}
University of Virginia School of Medicine
{Staff EMAIL}

Document S4. "Not High Genetic Risk" letter.

December 20, 2018

Parent/guardian of «Participant_First» «Participant_Last»
«Street_Address»«Apt_PO_Box»
«City», «State», «Zip»

Dear parent/guardian of «Participant_First» «Participant_Last»,

Thank you for participating in the research study, IRB-HSR#20939: The Virginia PrIMeD (Precision Individualized Medicine for Diabetes) Project: Genetic Risk of Type 1 Diabetes.

We have evaluated the saliva specimen that «Participant_First» provided during the study visit, and have performed the DNA test. Based upon current knowledge of the genetic contribution to risk of type 1 diabetes, our testing showed that «Participant_First» is ***not*** at highest genetic risk.

This genetic test was performed for research purposes and is not based on an FDA-approved clinical test.

What does this result mean?
Our study defines high genetic risk for type 1 diabetes as being within the highest 5% of genetic risk (the 95th percentile or higher). Your child's results indicate that he or she is not within the top 5% of genetic risk for type 1 diabetes as defined by this study.

While genetic risk contributes about 50% of the total risk of developing type 1 diabetes, approximately 50% of the overall risk is due to non-genetic or environmental causes. This means that individuals who are not at high genetic risk can still develop type 1 diabetes.

Thank you for participating in the study. Please contact us at {Study telephone number} or {Study email} with any questions or concerns.

Sincerely,

{Study staff member}
University of Virginia School of Medicine
{Study email address}

Document S5. Follow-up questionnaire.

FOLLOW-UP QUESTIONNAIRE

Name of interviewer _______________________(First, middle, last)

Today's date _____ _______ _________
MM DD YYYY

Study ID:

Participant's (child) full name: _________________________(First, middle, last)

Full name of parent/guardian completing questionnaire:

____(First, middle, last)

Age of child: ___________ (hand-enter #, must be between 0 and 18)

Review of Participant Contact Information

We would like to collect contact information for you and your child in order to share study information with you and, with your permission, to follow your child over time for future health outcomes related to type 1 diabetes.

Primary caretaker phone number

- ○ Home/cell (XXX) XXX-XXXX
- ○ Work (XXX) XXX-XXXX

Preferred phone contact: ☐ home/cell ☐ work

Primary caretaker mailing address (Street, Apt/PO Box, City, State, Zip Code)

____

Primary caretaker email address:

____ (force to have @ sign)
Note: This email address will be used to automatically email you a copy of the consent form

Health History

Now we'd like to ask some questions **about your child** who is participating in this study.

Child's date of Birth _____ _______ _________
MM DD YYYY

Sex ☐ Male ☐ Female

Now we'd like to ask you some questions about type 1 diabetes. Type 1 diabetes is usually diagnosed during childhood, and is also known as juvenile diabetes, childhood diabetes, or T1D.

First, we'd like to ask about the medical history of <u>your child's relatives</u> (family history).

Does the child have a family history of Type 1 diabetes? Please base your answer on blood relatives.

☐ Yes [If Yes, prompted to answer next question.]
☐ No
☐ Don't know

[This question is only prompted by people who answer "Yes" above to family history of diabetes]
Which of your child's family members has been diagnosed with type 1 diabetes? Please only respond based on blood relatives.

☐ mother ☐ father ☐ sibling ☐ other

Does the child have a <u>family history</u> of autoimmune disease, other than Type 1 diabetes? Please base your answer on blood relatives.

☐ Yes [If Yes, prompted to answer next question.]
☐ No
☐ Don't know

If yes, please mark all autoimmune conditions diagnosed among your child's blood relatives:

☐ Thyroid (Hashimoto and/or Graves)
☐ Celiac Disease
☐ Addison Disease
☐ Other (please list ______________________)
☐ Blood relative has been diagnosed with autoimmune disease, but I don't know which condition

Next, we'd like to ask about <u>your child's</u> medical history.

Has your medical provider ever told you that your child had high blood sugar?
☐ Yes ☐ No ☐ Don't know

Has your child ever been diagnosed with type 1 diabetes?
☐ Yes ☐ No ☐ Don't know
[If yes to above question about diabetes, prompt to answer the next four questions]

At what age was your child diagnosed with type 1 diabetes?
________________ (fill in, require # between 0-18)

Is your child currently on any medication for type 1 diabetes?
☐ Yes ☐ No ☐ Don't know

Has your child ever been hospitalized for type 1 diabetes?
☐ Yes ☐ No ☐ Don't know
[If yes, fill in month and year]

Has your child ever had diabetic ketoacidosis (DKA)?
☐ Yes ☐ No ☐ Don't know

Has your child ever been diagnosed with an autoimmune disease, other than Type 1 diabetes?
☐ Yes ☐ No ☐ Don't know

[If yes to above question about autoimmune disease, prompt to answer the next question]
If yes, please mark all autoimmune conditions:
☐ Thyroid (Hashimoto and/or Graves)
☐ Celiac Disease
☐ Addison Disease
☐ Other (please list __________________________)
☐ Child has been diagnosed with autoimmune disease, but I don't know which condition

Thank you for participating in The Virginia PrIMeD (Precision Individualized Medicine for Diabetes) Project: Genetic Risk of Type 1 Diabetes!

Fig S1. Study flyer.

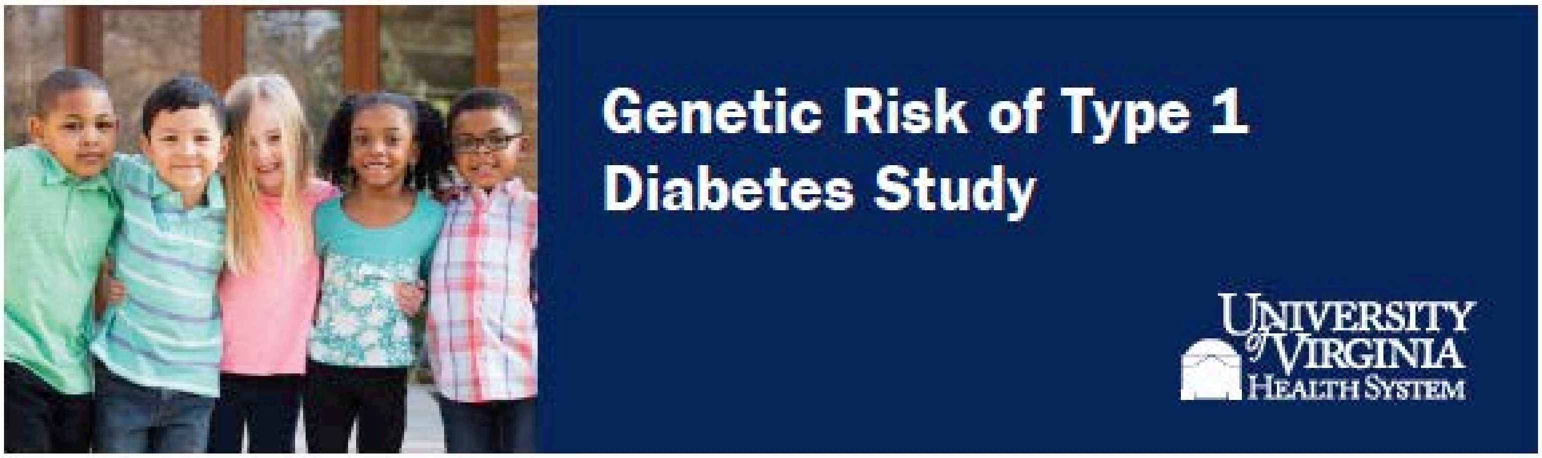

Genetic Risk of Type 1 Diabetes Study

UNIVERSITY of VIRGINIA HEALTH SYSTEM

Precision Individualized Medicine for Diabetes

Virginia PrIMeD Project

This clinic is participating in a study about type 1 diabetes, Precision Individualized Medicine for Diabetes – the Virginia PrIMeD Project. The University of Virginia study team is inviting children 2-16 years of age to participate in this study.

What is this study about?

This study will analyze variations in your child's genes, or DNA, to determine if they have a higher risk of developing type 1 or juvenile diabetes sometime in their lifetime. Variations in genes can identify about half of the risk of development of type 1 diabetes.

It is important to know that not everyone with high genetic risk of developing type 1 diabetes will actually develop the disease. And some people that do not have high genetic risk could possibly develop type 1 diabetes. The goal of this study is to identify children who might benefit from closer monitoring for and early education about type 1 diabetes.

This study will investigate whether early monitoring of children at high genetic risk of type 1 diabetes can improve outcomes for children who eventually develop the disease. In most cases, type 1 diabetes onset is sudden, with little or no warning. In about one-third of individuals with newly diagnosed type 1 diabetes, severe problems can occur. We are offering a way to try to prevent these complications.

If you agree to participate in this study, you/your child would be asked to:

- Complete a brief questionnaire
- Provide a saliva (spit) sample, which will be used to analyze your child's genes (DNA)
- Participate in brief annual follow-up contacts to update the questionnaire

After the initial study visit, the study team will:

- Study your child's genes (only genes related to type 1 diabetes risk)
- Determine whether your child is at high genetic risk for type 1 diabetes
- Communicate diabetes genetic risk results to you and your clinician

All study procedures are done at no cost to you, and are for research purposes only and separate from your medical care. We will not share this genetic risk information with your health insurance company or put this information into the medical record. However, you and your clinician may gain valuable information regarding your child's genetic risk of type 1 diabetes based on this research.

If you are interested in participating, the study team member at your clinic can discuss the study procedures and goals in more detail with you.

Whether you decide to participate, this study will have no impact on the medical care that you receive today.

Virginia Precision Individualized Medicine for Diabetes (PrIMeD) Project

Principal Investigator: Dr. Stephen S. Rich, PhD
434.982.3228
ssr4n@virginia.edu

UVA Center for Public Health Genomics
PO Box 800717 | Charlottesville, VA 22908

IRB-HSR #20023

Genetic Risk of Type 1 Diabetes Study

University of Virginia Health System

Frequently Asked Questions

If type 1 diabetes does not run in my family, does that mean that my child is at low genetic risk?
Most people (over 90%) who have type 1 diabetes do not have a family member with the disease. However, if your child has a close relative with type 1 diabetes, your child is more likely to have a high genetic risk of type 1 diabetes.

Does this test determine the genetic risk for type 2 diabetes?
No. The risk factors for adult onset (type 2) diabetes, are different from risk for type 1 diabetes and are not analyzed in this study.

How accurate are these genetic tests?
The accuracy of the genetic test is over 99% for analyzing the genes related to type 1 diabetes. However, the tests can only determine whether a child is at high risk of developing type 1 diabetes. It cannot tell who will and who will not develop type 1 diabetes. This is because half of the risk is due to other unknown factors.

What are the researchers looking for?
We will identify children at high genetic risk of type 1 diabetes so that your clinician can offer additional screening and close monitoring of your child over time. We will only look at genes related to risk of development of type 1 diabetes.

How will this study benefit my child?
You can benefit by knowing whether your child is at high risk for developing type 1 diabetes. If your child is at high risk, we may offer you entry into another study that will monitor children (at no cost to you) for early signs of type 1 diabetes. Type 1 diabetes usually appears suddenly, and it is the sudden onset that can be life-threatening. We are offering a way to learn what to look for, which may help with earlier diagnosis and result in less severe symptoms if your child does go on to develop type 1 diabetes.

Are you going to test for anything else at any time? How long will you keep my child's specimen?
We will only use the saliva (spit) sample to test for genetic markers related to risk for type 1 diabetes. We will not test for the risk of other diseases. The saliva sample will be discarded within a few weeks, soon after the genetic analysis is complete.

Will my child's health insurance be affected by these results?
We will not disclose the results of this test to your insurance company. If fact, study participants are protected by a Certificate of Confidentiality from the National Institutes of Health (NIH), which means that no one can force us to share the genetic information from this study with your insurance company.

Will I get the outcome of this test?
Yes, we will contact you within eight weeks of the study visit to inform you whether the genetic test shows high genetic risk of type 1 diabetes.

What if I don't want to know the results of my child's genetic testing?
We will follow up with patients at high genetic risk for monitoring and education. Therefore, if you do not want to know the results of your child's genetic testing, you should not participate in this study.

What happens after the genetic test result?
We will continue to follow up with all participants who agree to participate in this study, regardless of genetic risk score, on a yearly basis for a brief questionnaire on medical history.

For participants with high genetic risk scores, additional support will be available from the study team and your study-affiliated clinic. You/your child may be invited to participate in additional studies related to type 1 diabetes.

18148221, 1/18

Virginia Precision Individualized Medicine for Diabetes (PrIMeD) Project

Principal Investigator: Dr. Stephen S. Rich, PhD
434.982.3228
ssr4n@virginia.edu

UVA Center for Public Health Genomics
PO Box 800717 | Charlottesville, VA 22908

IRB-HSR #20023

Table S1. T1D genetic risk score SNP list.

SNP	Effect Allele	Effect Allele Frequency	Weight	Chromosome	Position	Other Allele
rs2476601	A	0.09633	0.69445	1	114377568	G
rs6691977	C	0.19504	0.15864	1	200814959	T
rs3024505	T	0.16084	-0.19027	1	206939904	C
rs13415583	G	0.35891	-0.10278	2	100764087	T
rs4849135	T	0.29009	-0.11164	2	111615079	G
rs2111485	A	0.39300	-0.17099	2	163110536	G
rs35667974	G	0.02198	-0.66208	2	163124637	A
rs35337543	C	0.01471	-0.42256	2	163136505	G
rs3087243	A	0.45189	-0.18031	2	204738919	G
rs113010081	C	0.11820	-0.17816	3	46457412	T
rs2611215	T	0.15176	0.18345	4	166574267	C
rs2194225	G	0.39836	0.10113	5	35883804	A
rs6906897	T	0.02772	0.46444	6	29415636	C
rs2523409	C	0.36905	0.18261	6	29775662	T
rs3094165	T	0.28372	-0.03334	6	29833541	C
rs1150743	A	0.09473	0.32150	6	29992261	G
rs28732101	T	0.05480	-1.17270	6	31106302	C
rs3130933	A	0.13865	-0.69673	6	31132085	G
rs2524089	C	0.42497	0.06771	6	31266522	A
rs2256974	T	0.17826	0.20223	6	31555392	G
rs436845	C	0.24835	-0.79953	6	32197736	T
rs6935715	G	0.00589	-0.19284	6	32288238	T
rs2143461	T	0.19483	0.45090	6	32335347	C
rs2076531	C	0.00398	-1.47029	6	32363712	T
rs3763305	A	0.05453	0.21831	6	32369488	G
rs9268633	A	0.19924	-2.61771	6	32406473	G
rs6903608	C	0.27034	-1.15729	6	32428285	T
rs9271366	G	0.14454	-3.34384	6	32586854	A
rs9273363	A	0.30135	1.68202	6	32626272	C
rs1049225	T	0.23046	-1.76991	6	32627747	C
rs9357152	G	0.24373	-1.13995	6	32664960	A
rs3129722	A	0.02469	-2.45039	6	32676343	C
rs7745656	T	0.25186	-1.31359	6	32680970	G
rs2239800	C	0.08263	-1.18291	6	32713267	T
rs2071463	A	0.15341	-0.74812	6	32812528	G
rs635688	T	0.41185	-0.53403	6	32943151	C
rs1367728	A	0.10918	-0.67613	6	33034815	G

rs9469341	G	0.18272	-0.15072	6	33035877	A
rs72928038	A	0.17683	0.18426	6	90976768	G
rs2045258	C	0.44860	0.12383	6	126674354	T
rs62447205	G	0.28133	-0.14080	7	50465830	A
rs7780389	T	0.04774	-0.34152	7	51015193	C
rs1574285	G	0.40208	0.10273	9	4283137	T
rs61839660	T	0.10323	-0.49665	10	6094697	C
rs10795791	G	0.41228	0.18220	10	6108340	A
rs41295121	T	0.01025	-0.68209	10	6129643	C
rs12416116	A	0.29014	-0.17929	10	90035654	C
rs689	T	0.29147	-0.83247	11	2182224	A
rs72853903	T	0.30592	-0.32124	11	2198665	C
rs917911	G	0.36082	0.11741	12	9905851	T
rs705704	A	0.33974	0.21566	12	56435412	G
rs653178	A	0.48704	-0.27527	12	112007756	G
rs9585056	C	0.24820	0.16031	13	100081766	T
rs1456988	G	0.27724	0.11527	14	98488007	T
rs56994090	C	0.41329	-0.13275	14	101306447	T
rs72727394	T	0.19982	0.14186	15	38847022	C
rs34593439	A	0.10610	-0.28229	15	79234957	G
rs12927355	T	0.31999	-0.20969	16	11194771	C
rs193778	C	0.25834	0.13483	16	11351211	T
rs151233	A	0.12951	0.20043	16	28506428	G
rs757411	C	0.36619	-0.10952	17	38775150	T
rs1052553	G	0.24119	-0.11739	17	44073889	A
rs1893217	C	0.16923	0.24616	18	12809340	T
rs12971201	A	0.39879	-0.16778	18	12830538	G
rs1615504	A	0.47244	0.12369	18	67526644	G
rs34536443	C	0.04864	-0.44129	19	10463118	G
rs12720356	G	0.09680	-0.16714	19	10469975	T
rs402072	G	0.16058	-0.14680	19	47219122	A
rs516246	G	0.49363	-0.14021	19	49206172	A
rs6043409	A	0.35031	-0.13008	20	1616206	G
rs11203203	A	0.36656	0.16174	21	43836186	G
rs6518350	G	0.18522	-0.11173	21	45621817	A
rs4820830	C	0.38785	0.15980	22	30531091	T
rs229533	C	0.43331	0.13488	22	37587111	A
